# A Four-Pseudogene Classifier Identified by Machine Learning Serves as a Novel Prognostic Marker for Survival of Osteosarcoma

**DOI:** 10.3390/genes10060414

**Published:** 2019-05-29

**Authors:** Feng Liu, Lu Xing, Xiaoqian Zhang, Xiaoqi Zhang

**Affiliations:** 1 Shandong Provincial Key Laboratory of Oral Tissue Regeneration, School of Stomatology, Shandong University, Jinan 250014, Shandong, China; ed_liufeng@163.com (F.L.); xinglu4@126.com (L.X.); 2 Department of Stomatology, Haiyuan College of Kunming Medical University, Kunming 650000, Yunnan, China; zhangxiaoqian817@163.com

**Keywords:** noncoding RNA, pseudogene, biomarker, prognosis, survival, machine learning, osteosarcoma

## Abstract

Osteosarcoma is a common malignancy with high mortality and poor prognosis due to lack of predictive markers. Increasing evidence has demonstrated that pseudogenes, a type of non-coding gene, play an important role in tumorigenesis. The aim of this study was to identify a prognostic pseudogene signature of osteosarcoma by machine learning. A sample of 94 osteosarcoma patients’ RNA-Seq data with clinical follow-up information was involved in the study. The survival-related pseudogenes were screened and related signature model was constructed by cox-regression analysis (univariate, lasso, and multivariate). The predictive value of the signature was further validated in different subgroups. The putative biological functions were determined by co-expression analysis. In total, 125 survival-related pseudogenes were identified and a four-pseudogene (RPL11-551L14.1, HR: 0.65 (95% CI: 0.44–0.95); RPL7AP28, HR: 0.32 (95% CI: 0.14–0.76); RP4-706A16.3, HR: 1.89 (95% CI: 1.35–2.65); RP11-326A19.5, HR: 0.52(95% CI: 0.37–0.74)) signature effectively distinguished the high- and low-risk patients, and predicted prognosis with high sensitivity and specificity (AUC: 0.878). Furthermore, the signature was applicable to patients of different genders, ages, and metastatic status. Co-expression analysis revealed the four pseudogenes are involved in regulating malignant phenotype, immune, and DNA/RNA editing. This four-pseudogene signature is not only a promising predictor of prognosis and survival, but also a potential marker for monitoring therapeutic schedule. Therefore, our findings may have potential clinical significance.

## 1. Introduction

Osteosarcoma is the most common malignancy derived from bone, which is originated in mesenchymal tissue like many other sarcomas. It usually occurs in children and young adults as well as the elderly with a typical bimodal distribution in age [1]. The incidence of osteosarcoma is 4.4 per-million among those aged 0–24 years old, and in the second peak of age the disease is usually secondary, accompanied with Paget’s disease or other bony lesions [2]. The primary therapy for osteosarcoma is surgery, but the survival of patients with treatment of surgery alone remains disappointing, around 15–17% [3]. At present, the therapy for osteosarcoma is a combination of chemotherapy and surgery, which could cure about 70% of the patients. For the patients with localized tumor, the reaction of chemotherapy is the best predictor to predict prognosis for now [4]. However, for patients with recurrent or metastatic disease, their overall survival is still not optimistic, which remained at 20% for the last 30 years [5]. Notwithstanding in the advances in surgical techniques, targeted therapy, and tumor immunity, the complications involved with infection and inconvenience resulting from limb-salvage surgery as well as the low survival rates makes it urgent to develop prediction methods for the improvement of survival of osteosarcoma patients [2]. Prognostic biomarkers can provide information about the probable outcome of a cancer relative to disease progression, recurrence, or death [6]. This could provide considerable help with patient stratification, treatment management, and monitoring disease status in clinical practice, such as offering personalized therapeutic schedules for osteosarcoma patients which would benefit enormously [7]. Therefore, it will be very helpful for treatments of osteosarcoma if accompanied by suitable prognostic prediction.

Pseudogenes are a class of homologues of the corresponding functional genes, which are also known as ‘gene fossils’ or ‘junk genes’. They belong to a subclass of long non-coding RNAs [8]. Their inability in expressing functional proteins was because of various mutations in their coding sequences including deletions, insertions, frameshift mutations, etc., which often lead to premature termination of codons [9]. However, pseudogenes can still have numerous regulatory functions by being transcribed into small interference RNAs (siRNAs) [10], competitive endogenous RNA (ceRNA) [11], antisense transcripts [12], and sequestering miRNAs [13]. Studies have shown that aberrant expression of pseudogenes participate in many diseases including cancer [14]. Some cancer related pseudogenes could regulate the expression of their corresponding coding genes such as KRAS and KRASP1, KRAS, and KRASP1 by sequestering the interacting miRNAs [13]. Besides, studies have found aberrant expression of the pseudogenes of transcription factors is critical for maintaining embryonic stem cell pluripotency (i.e., NANOG and NONOGP1, POU5F1P1, and OCT4) in cancer [15]. Some cancer related pseudogenes can be used as biomarkers for prognosis. In hepatocellular carcinoma, high expression level of the pseudogene RP11-564D11.3 is observed to be related with poor prognosis [16], and in another study conducted by Ganapathi et al., they found that pseudogene SLC6A10P can work as a predictive marker for recurrence in high-grade ovarian cancer [17]. However, due to lack of attention and limited number of samples, the potential of pseudogenes as biomarkers for prognosis in osteosarcoma has been not studied.

Recent development in bioinformatics and the availability of large-scale RNA-seq transcriptome data of multiple cancers with clinical follow-up data provide better approaches to explore the biomarkers for diagnosis and prognosis, allowing for a better understanding of the mechanism of cancer and improvement for patients’ outcome [18]. However, most of these studies aimed to construct a diagnosis or prognosis signature are mainly focused on genes, lncRNAs, miRNAs, DNA methylation, as well as alternative splicing [18,19,20,21,22], and pseudogenes’ potential as biomarker has been neglected in osteosarcoma, despite aberrant expression of pseudogenes have been found to be related to multiple pathological processes in cancer and work as a promising biomarker in other types of cancers [23].

In our study, we applied machine learning analysis including univariate cox regression, LASSO cox regression, and multivariate cox regression analysis to construct a pseudogene-based signature to predict the prognosis outcome for osteosarcoma patients. First, by univariate regression, we identified survival related pseudogenes. Next, we narrow down the significantly prognosis related pseudogenes by LASSO regression and multivariate regression, from which we constructed a four-pseudogene based prognostic signature. Then we assessed the clinical utility of this prognostic model and explored its potential functions. Our findings provide new insights into predicting and evaluating the clinical outcome of osteosarcoma patients.

## 2. Materials and Methods

### 2.1. Data Acquisition

The osteosarcoma RNA sequencing (RNA-seq) expression data and the corresponding clinical follow-up data were obtained from the public database TARGET (https://ocg.cancer.gov/). In total, there are clinical information of 274 patients and RNA-seq data of 101 patients. TPM is used in this study which is a kind of RNA-seq data scaled by gene length and sequencing depth (M) [24]. After excluding the samples with incomplete information, as well as pseudogenes with low expression (average TPM ≤ 1 across all samples), 94 osteosarcoma expression data of 1333 pseudogenes accompanied by corresponding clinical follow-up information was obtained. The expression value was processed as log2 (TPM + 1) for further analysis [25].

### 2.2. Construction of Prognostic Signature

Univariate cox regression analysis was performed to screen survival related pseudogenes. The pseudogenes with *p*-value < 0.05 were screened as candidate pseudogenes for next analysis (FDR for *p*-value adjustment). These candidate pseudogenes were further screened by LASSO regression, which is an efficient method for regression analysis of high-dimensional predictors [26], and was widely used in Cox proportional hazard regression model for survival analysis [27]. 10-fold cross-validation was conducted to select the tuning parameter to determine the magnitude of penalization, and was then used to choose stable features with nonzero coefficient [28,29,30,31,32]. The coefficients of screened pseudogenes are as following: RP11_551L14.1 (−0.0943699924), RPL7AP28 (−0.1729184112), SRSF9P1 (−0.0077455177), ZC3H11B (0.0326745474), RP4_706A16.3 (0.3262156868), AC079781.5 (0.0161718261), NBPF2P (0.1020104257), TMSB10P2 (−0.0997438836), ZNF815P (−0.0104546653), SLC25A24P1 (0.0271387454), CTA_963H5.5 (−0.0131888444), RP11_241F15.10 (−0.0008593915), RP11_23J18.1 (−0.0168592205), RP11_326A19.5 (−0.2405703963), RP11_344N17.15 (−0.0439379021). Then, multivariate regression analysis was performed and pseudogenes with *p* < 0.05 was selected for predictive signature construction. Corresponding risk score of each patient was calculated by the formula: risk score = expression of RPL11-551L14.1 × β (−0.4327) + expression of RPL7AP28 × β (−1.1344) + expression of RP4_706A16.3 × β(0.6360) + expression of RP11_326A19.5 × β (−0.6503). Patients were divided into high and low risk groups according to the median risk score (1.23). The Kaplan–Meier (K–M) survival analysis was performed on the two groups. Furthermore, the receiver operating characteristic (ROC) analysis was performed to evaluate the predicting efficiency of the model to predict 3-, 5-, and 8-year survival and the area under curve (AUC) was calculated. All of these processes were conducted by R software (version 3.5.1).

### 2.3. Nomogram and Calibration

We combined several basic clinical information with the risk score signature to predict the 3-, 5-, and 8-year survival of osteosarcoma patients by plotting nomogram. Calibration plot, which demonstrates whether the predicted outcome is similar with the actual outcome, was used to evaluate the performance of the nomogram. In this section, R package rms was used to plot nomogram and calibration plot.

### 2.4. Correlation Analysis of the Four Pseudogenes and Annotation of Their Function

The Pearson correlation coefficients between the expression profiles of the four prognostic pseudogenes and protein-coding genes (PCGs) were calculated to determine the co-expression relationships of the pseudogenes and PCGs. The PCGs positively or negatively correlated with the four pseudogenes were considered as pseudogene-related PCGs for functional analysis. The online Database for Annotation, Visualization, and Integrated Discovery (DAVID, https://david.ncifcrf.gov/summary.jsp) [33] was used to explore the functions of pseudogenes-related PCGs and *p*-value < 0.05 was regarded significant, which is described in detail in our previous studies [19,21,34,35]. The biological processes and KEGG (Kyoto Encyclopedia of Genes and Genomes) pathways were presented.

## 3. Results

### 3.1. Clinical Characteristics of the Patients

**Figure 1:**
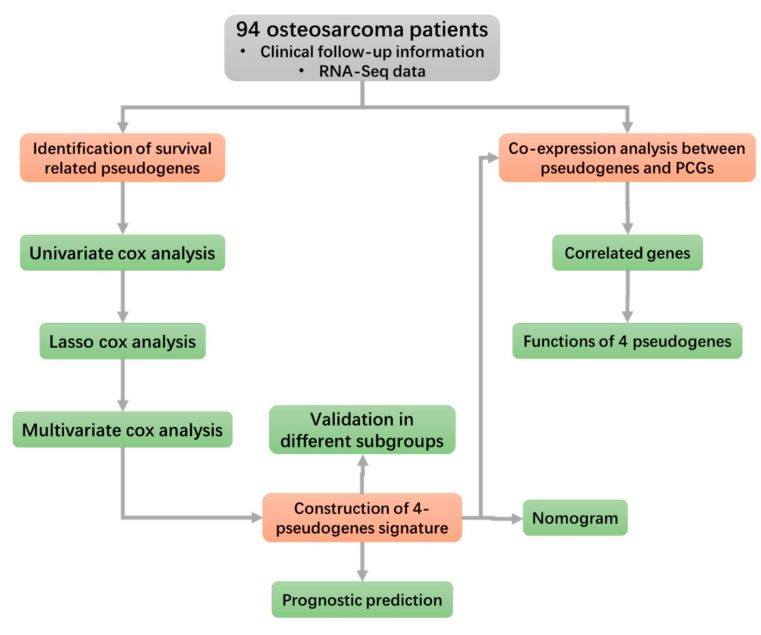
Flowchart of this study. It exhibits the methods and processes of this study to make it easier for readers to have a better overview of it.

The osteosarcoma cohort includes data from a total of 274 samples. After excluding samples without clinical follow-up information, 94 osteosarcoma patients were involved in this study and the baseline of clinical characters were presented (Table 1). The study flowchart is outlined in Figure 1.

### 3.2. Identification of Osteosarcoma Survival-Related Pseudogenes

**Figure 2:**
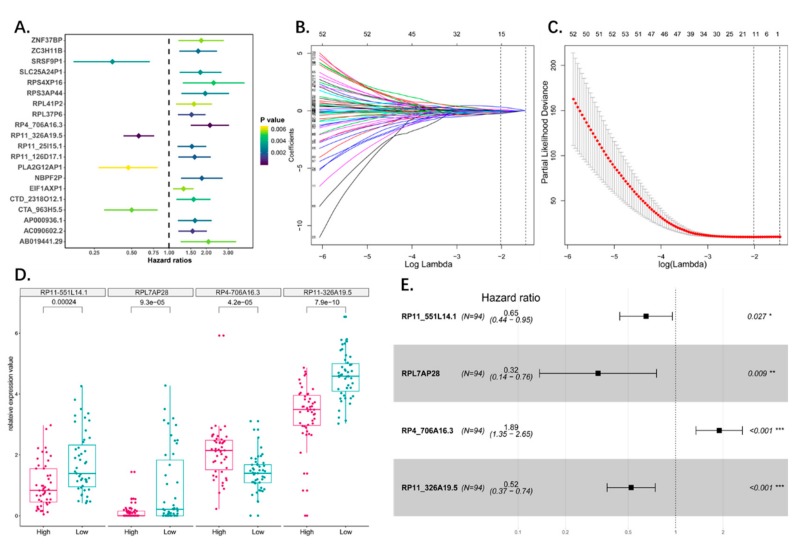
(**A**) The forest plot showed the top 20 (20/125) significant survival related pseudogenes through univariate cox analysis. (**B**) LASSO coefficient profiles of the survival related pseudogenes. A coefficient profile plot was produced against the log lambda sequence. Vertical line was drawn at the value selected using 10-fold cross-validation, where optimal lambda resulted in 15 nonzero coefficients. (**C**) Tuning parameter (lambda) selection in the LASSO model used 10-fold cross-validation via minimum criteria. Dotted vertical lines were drawn at the optimal values by using the minimum criteria and the 1 standard error of the minimum criteria (the 1-SE criteria). A lambda value of 0.13 was chosen (lambda.min) according to 10-fold cross-validation. (**D**) Boxplot showed the expression status of the four pseudogenes between high risk and low risk group which divided by median risk score. (**E**) Forest plot showed results of multivariate cox analysis. Four significant pseudogenes in multivariate cox analysis were screened out (*p*-value < 0.05) as candidate to construct the risk model.

The survival-related pseudogenes were screened using univariate Cox proportional hazards regression analysis. Using *p* < 0.05 as the cut off, 125 pseudogenes were identified by the univariate analysis as significant to overall survival (Table 2 and Table S1). The top 20 significant survival-related pseudogenes were demonstrated by Forest plot (Figure 2A).

### 3.3. Construction of the Prognostic Pseudogene Signature for Osteosarcoma

The predictive model was constructed based on the 125 survival-related pseudogenes using LASSO regression. The LASSO Cox regression model was used to select variables in order to avoid overfitting of the predictive model, and 15 pseudogenes with non-zero coefficient were selected with the minimum criteria (Figure 2B,C). Multivariate Cox proportional hazards regression analysis was then performed, and the significant pseudogenes (*p* < 0.05) were used to construct the model. A four-pseudogene (RPL11-551L14.1, RPL7AP28, RP4-706A16.3, and RP11-326A19.5) based risk model was finally obtained (Figure 2E; Table 3), and a risk-score formula was established according to their expression levels. The four-pseudogene risk score for each patient was calculated, and the patients were divided into high and low risk groups (*N* = 47 each) according to the median risk score (Figure 2D; Table 4, Table S2).

### 3.4. Predictive Value of the Four-Pseudogene Signature

**Figure 3:**
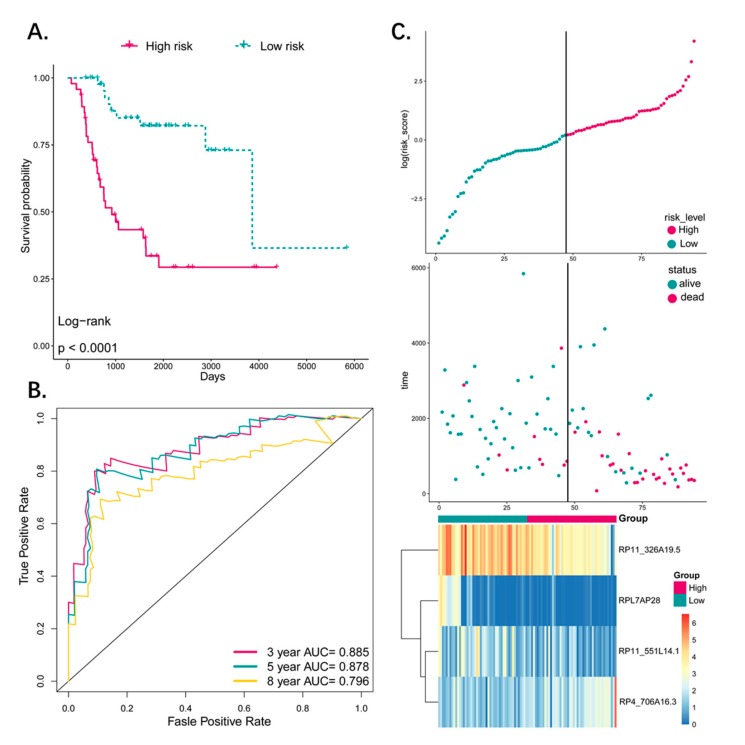
(**A**) The Kaplan–Meier estimates of the OS for high-risk and low-risk patient cohorts grouping by the four-pseudogene signature (*N* = 94). The OS differences between the two groups were determined by the two-sided log-rank test. It can be concluded that higher risk scores are significantly associated with worse OS (*p* < 0.0001). (**B**) ROC analysis of sensitivity and specificity for the four-pseudogene signature in predicting the OS of patients for 3-, 5-, and 8-years. (**C**) The distribution of four-pseudogene risk score, patients’ survival status and pseudogene expression signature were analyzed. As the risk score rising, the patients had a shorter survival time, more death events and the expression value of four pseudogenes ascended or decreased. OS: overall survival.

Kaplan–Meier survival analysis was performed to assess the potential prognostic value of the four-pseudogene signature, which revealed significantly worse prognosis of the patients in the high-risk group (*p* < 0.0001; Figure 3A). In addition, ROC analysis was used to determine the accuracy of this signature in predicting the 3-, 5-, and 8-year survival (Figure 3B), and the respective AUCs were 0.885, 0.878, and 0.796, indicating high sensitivity and specificity of this signature. In addition, a higher risk score was associated with shorter survival and more death events (Figure 3C). Taken together, this four-pseudogene signature-based risk model can distinguish the high-risk osteosarcoma patients from the low-risk patients, indicating a prognostic significance for osteosarcoma.

### 3.5. The Four-Pseudogene Signature Is an Independent Prognostic Predictor of Osteosarcoma

**Figure 4:**
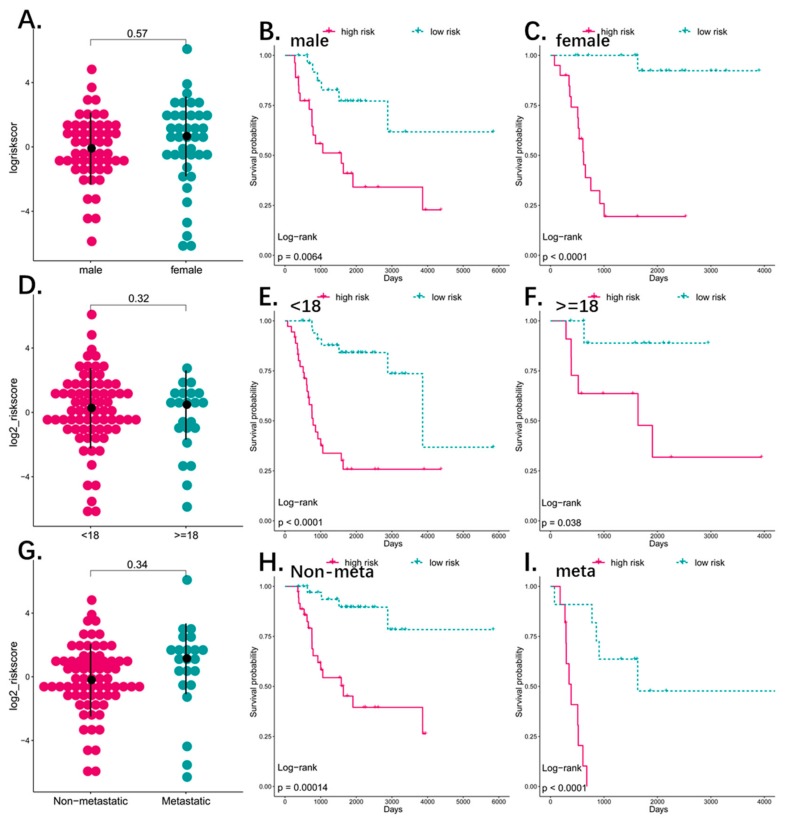
(**A**) The dot plot showed the distribution of risk score based four-pseudogene between male (*N* = 54) and female (*N* = 40). The risk score is not different between male and female (*p*-value > 0.05). (**B**,**C**) Kaplan–Meier analyses of patients with osteosarcoma in different gender cohorts, grouping based on their gender: male (*N* = 54), female (*N* = 40). Kaplan–Meier analysis with two-sided log-rank test was performed to estimate the differences in OS between the low-risk and high-risk patients in male cohort and female cohort. (**D**) The dot plot showed the distribution of risk score based four-pseudogenes between different age group (<18 and ≥18). The risk score is not different between the two groups (*p*-value > 0.05). (**E**,**F**) Kaplan–Meier analyses of patients with osteosarcoma in different age cohorts, grouping based on their age at initial diagnosis: <18 (*N* = 72), ≥18 (*N* = 22). Kaplan–Meier analysis with two-sided log-rank test was performed to estimate the differences in OS between the low-risk and high-risk patients in <18 and ≥18. (**G**) The dot plot showed the distribution of risk score based four-pseudogenes between different metastatic status group (non-metastatic = 72 and metastatic = 22). The risk score is not different between the two groups (*p*-value > 0.05). (**H**,**I**) Kaplan–Meier analyses of patients with osteosarcoma in different metastatic status cohorts. Kaplan–Meier analysis with two-sided log-rank test was performed to estimate the differences in OS between the low-risk and high-risk patients in metastatic and non-metastatic groups.

**Figure 5:**
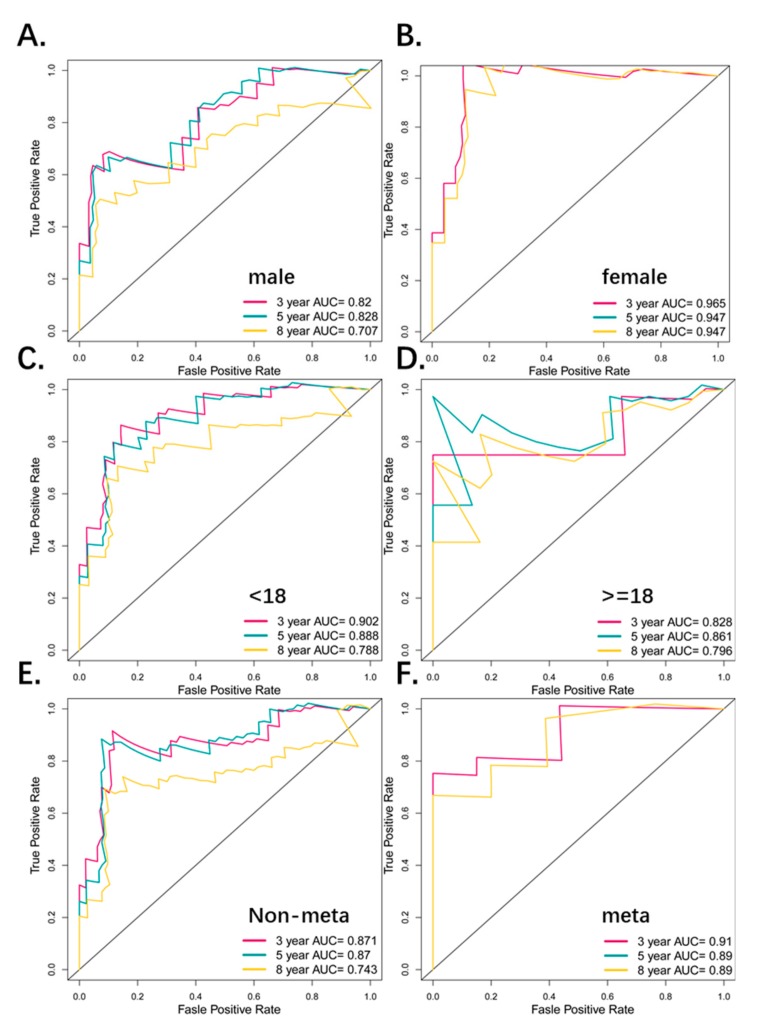
(**A**,**B**) ROC analyses of sensitivity and specificity for the four-pseudogene signature in predicting the OS of patients for 3, 5, and 8 years in the gender groups. (**C**,**D**) ROC analyses of sensitivity and specificity for the four-pseudogene signature in predicting the OS of patients for 3, 5, and 8 years in the age groups. (**E**,**F**) ROC analyses of sensitivity and specificity for the four-pseudogene signature in predicting the OS of patients for 3, 5, and 8 years in the metastatic groups.

To detect the possible contribution of other factors like age, gender, and metastatic status on patient survival, we also grouped the patients according the above variables, and applied the four-pseudogene signature to different sub-groups. There were 40 males and 54 females in the osteosarcoma cohort, which did not differ significantly in terms of the risk score distribution (Figure 4A). In addition, the high-risk patients had significantly shorter overall survival (OS) (*p* < 0.01) in both the male and female groups (Figure 4B,C) with respective five-year AUC values of 0.753 and 0.805 (Figure 5A,B), indicating that the four-pseudogene signature was independent of gender. There was also no significant difference in the distribution of the risk score of younger (<18 years, *N* = 72) and older (>18 years, *N* = 22) patients (Figure 4D). Furthermore, the low-risk patients had significantly longer OS (*p* < 0.01; Figure 4E,F), and similar five-year AUC values (0.888 and 0.861; Figure 5C,D) in both age-stratified groups, indicating that the four-pseudogene signature was also independent of age. Based on metastatic status, the patients were divided into non-metastatic and metastatic groups. The both have a similar risk score and a shorter OS in high risk group (Figure 4G–I), and the five-year AUC values were 0.87 and 0.89 for non-metastatic and metastatic respectively (Figure 5E,F), indicating that the four-pseudogene signature was independent of metastatic status. Taken together, the four-pseudogene signature can be applied to osteosarcoma patient subgroups stratified on the basis of clinical characteristics, and is an independent prognostic predictor for osteosarcoma.

### 3.6. Combined Pseudogene and Clinical Risk Score Is an Independent Predictor of Survival

**Figure 6:**
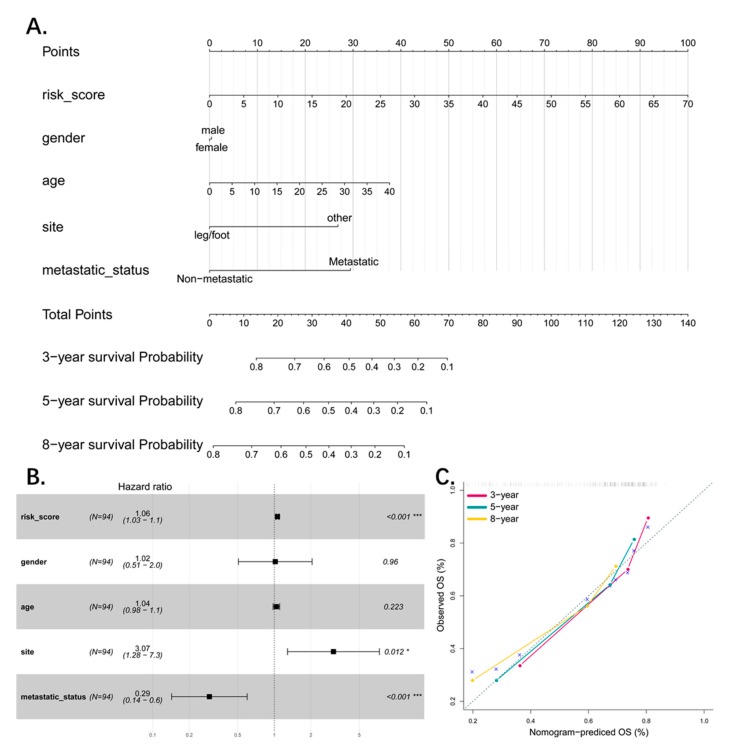
(**A**) Nomograms combining 4-pseudogene signature and clinical information to predict 3-, 5-, and 8-year survival probability of patients. (**B**) Forest plot showed results of multivariate cox analysis with 4-pseudogene signature and clinical information. The 4-pseudogene signature is an independent factor (*p*-value < 0.001). (**C**) The calibration of each model in terms of agreement between predicted and observed 3-, 5-, or 8-year outcomes. Model performance is shown by the plot, which is highly relative to the 45-degree line, representing perfect prediction.

To combine the basic clinical status (age, gender, site, and metastatic status) with the four-pseudogene signature for predicting patient survival, we constructed a multivariable cox probability hazard model to predict the 3-year, 5-year, and 8-year survival, and visualized it by a nomogram with an assigned score for each term (Figure 6A). Both the forest plot and the nomogram indicated that the risk score is an independent predictor for patient survival (Figure 6B). We also tested the stability and accuracy of the nomogram in terms of agreement between prediction and actual survival. The model performance is shown in Figure 6C and represents perfect prediction.

### 3.7. Functional Analysis of the Predictive Pseudogenes

**Figure 7:**
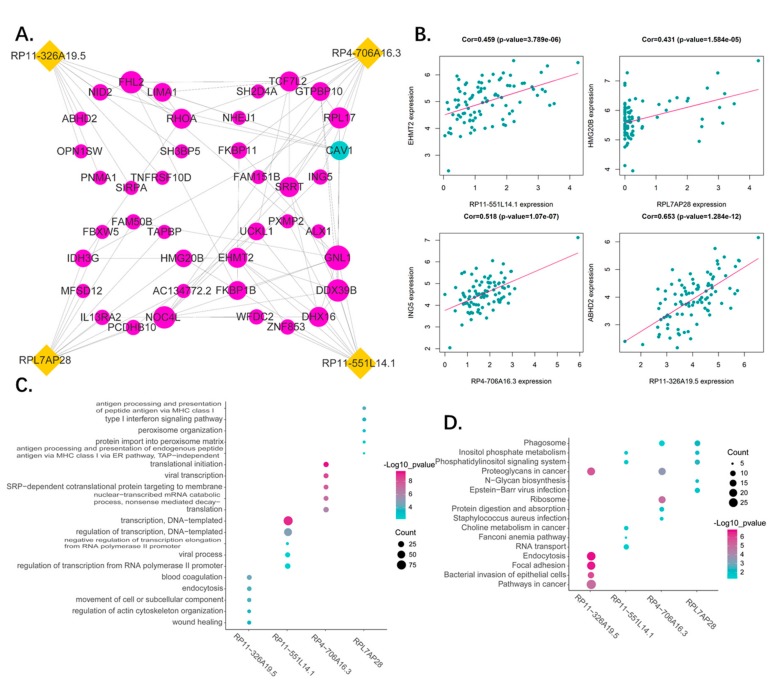
(**A**) Network of 4 pseudogenes and their top 10 correlated genes. The correlation between pseudogenes and genes were performed by Pearson correlation analysis. The interaction among genes were generated by STRING database. Yellow dots represent pseudogenes, red dots present positive corelated genes and blue dots represent negative corelated genes. The dot size represents node degree. (**B**) The representative of corelated genes. The dot plots show the most correlated genes of each pseudogenes. (**C**) The gene ontology enrichment analysis for the four pseudogenes correlated genes were carried out in DAVID to reveal the potential function of the four pseudogenes. The top five significant biological process terms for each pseudogene were shown. (**D**) The KEGG pathway enrichment analysis for the four pseudogenes correlated genes were carried out in DAVID to reveal the potential pathways in which the four pseudogenes are involved. The top five significant pathway terms for each pseudogene were shown.

Correlation analysis was used to identify the protein coding genes co-expressing with the pseudogenes, followed by gene ontology (GO) and Kyoto Encyclopedia of Genes and Genomes (KEGG) enrichment analysis to determine their function. The top 10 correlated PCGs of each pseudogene and their relationship are presented as a network (Figure 7A). The genes with highest correlation to each pseudogene were as follows: RPL11-551L14.1—EHMT2, RPL7AP2 8—HMG20B, RP4-706A16.3—ING5, and RP11—326A19.5—ABHD2 (Figure 7B). The functions of the pseudogenes as per GO (BP, biological process) and KEGG analyses are shown in Figure 7C,D. We found RP11-326A19.5 is associated with cell migration and adhesion such as wound healing, focal adhesion, and pathways in cancer. The RPL11-551L14.1 is participating in regulation of transcription and molecular metabolism. As for RP4-706A16.3, it plays a role in translation of ribosome. Lastly, RPL7AP28 may be related to immune regulation of MHC. A simple summary table for all results was in Table 5.

### 3.8. Comparison and Combination of Gene-Signature and Pseudogene-Signature

**Figure 8:**
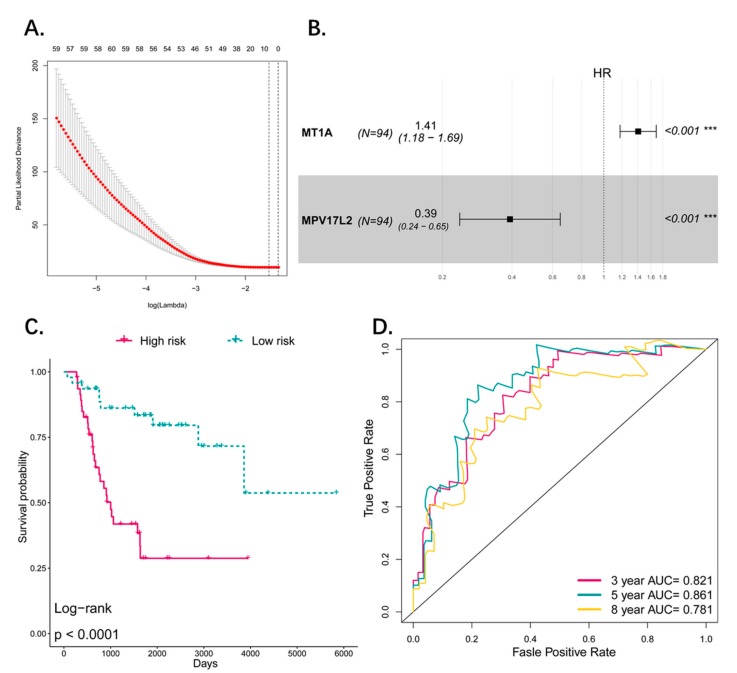
(**A**) Tuning parameter (lambda) selection in the LASSO model used 10-fold cross-validation via minimum criteria. Dotted vertical lines were drawn at the optimal values by using the minimum criteria and the 1 standard error of the minimum criteria (the 1-SE criteria). A lambda value of 0.22 was chosen (lambda.min) according to 10-fold cross-validation and 8 genes were identified for further analysis. (**B**) Forestplot showed results of multivariate cox analysis. Two significant coding genes in multivariate cox analysis were screened out (*p*_value < 0.05) as candidate to construct the risk model. (**C**) The Kaplan–Meier estimates of the OS for high-risk and low-risk patient cohorts grouping by the two-coding gene signature (*N* = 94). The OS differences between the two groups were determined by the two-sided log-rank test. It can be concluded that higher risk scores are significantly associated with worse OS (*p* < 0.0001). (**D**) ROC analysis of sensitivity and specificity for the two-gene signature in predicting the OS of patients for 3, 5, and 8 years.

**Figure 9:**
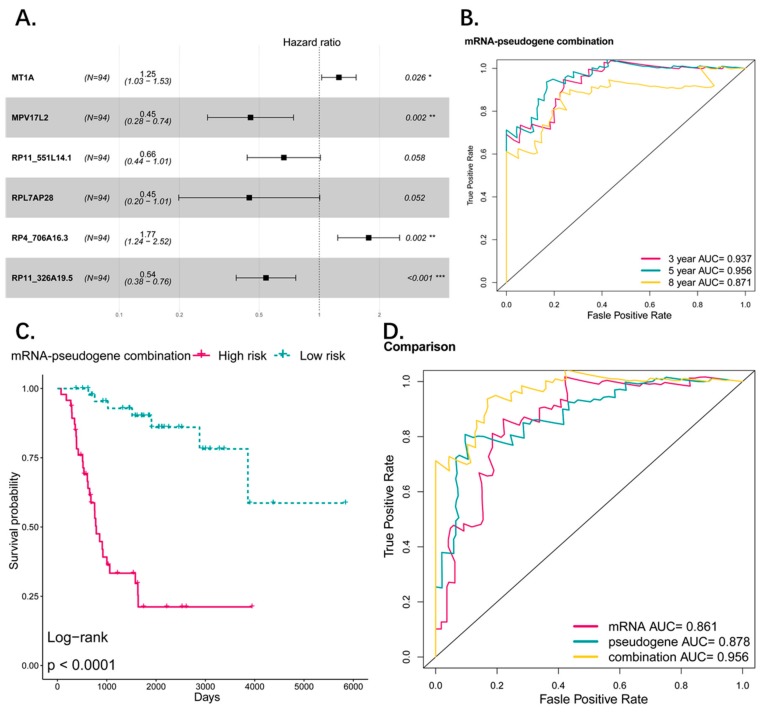
(**A**) Forest plot showed results of multivariate cox analysis for the combination model of genes and pseudogenes. (**B**) ROC analysis of sensitivity and specificity for the combination signature in predicting the OS of patients for 3, 5, and 8 years. (**C**) The Kaplan–Meier estimates of the OS for high-risk and low-risk patient cohorts grouping by the combination signature (*N* = 94). The OS differences between the two groups were determined by the two-sided log-rank test. It can be concluded that higher risk scores are significantly associated with worse OS (*p* < 0.0001). (**D**) ROC analysis of sensitivity and specificity for the comparison among the two-gene signature, four-pseudogene signature, and combination signature in predicting the OS of patients for 5 years and combination signature performed better than the other two.

To compare the performance of coding genes as signature with pseudogenes, we used the same method listed in the manuscript, and obtained a two-gene signature predicting the prognosis of osteosarcoma patients (MT1A, HR: 1.41 (95% CI: 1.18–1.69); MPV17L2, HR: 0.39 (95% CI: 0.24–0.65)) (Figure 8A,B). The Kaplan–Meier showed that the two-gene signature can distinguish high risk and low risk patients efficiently. The OS difference between the two groups was significant and high-risk group was associated with worse OS (*p* < 0.0001) (Figure 8C). ROC analysis revealed that the AUC of the signature predicting the 3-, 5-, and 8-year survival are 0.821, 0.861, and 0.781 respectively (Figure 8D). Compared with the pseudogene model (3-, 5-, and 8-year AUCs were 0.885, 0.878 and 0.796), it seems that the pseudogene-based model has a better performance in our study. Additionally, we combined the gene signature and pseudogene signature together to explore whether the pseudogenes can provide an additional power to the prediction of gene signature or they can work together to give a better performance than alone (Figure 9A). The ROC curve and K–M plot showed that the combined signature can also distinguish the high-risk and low-risk patients and the performance of predicting prognosis is satisfactory (Figure 9B,C). Comparing the three signatures by AUC of ROC curve, the combined signature performed best in sensitivity and specificity in which the AUC is 0.956 (5 years), indicating the genes and pseudogenes can work together to make a better prediction (Figure 9D).

## 4. Discussion

With the application of chemotherapy in the 1970s, the treatment of osteosarcoma has made great progress. However, survival rate of metastatic and relapse cases remains to be unsatisfactory, and the poor prognosis of such patients is the major problem for osteosarcoma [36]. Thus, identification of novel biomarkers to predict patients’ outcome might help to customize more personalized therapy and would be able to improve their prognosis. Growing evidence supports the role of pseudogenes in the oncogenesis and progression in different cancers [23,37,38] and there are also a few studies which recognized the importance of pseudogenes in osteosarcoma [39,40]. High throughput RNA-seq has paved the way for exploitation of various biomarkers for the diagnosis and prognosis of many cancers including osteosarcoma [18,41,42]. In this study, we took a systematic analysis for the potential role of pseudogenes as prognostic predictor and provided first evidence of survival related pseudogenes of osteosarcoma. We made several important discoveries during the course of this analysis. First, we identified 125 survival-related pseudogenes using univariate Cox analysis, and most of them are risk factors (91/125) which may play the oncogenesis role. Second, we identified a four-pseudogene signature and established a scoring system that was significantly associated with the OS of osteosarcoma patients. This signature helped to stratify the low- and high-risk groups and predicted the OS of osteosarcoma patients with high sensitivity and specificity. Out of four, RP4-706A16.3, is a risk factor and the another three are protective factors. Of the four pseudogenes we have identified, none were reported before, suggesting that these pseudogenes were newly found and required more attention. Third, in order to validate the applicability in different patients and extend the signature to various subgroups, K–M survival analysis and ROC curve analysis were performed in different subgroups. We found that it was independent of other potential predictors—including age, gender, and metastatic status—and the performance of predicting survival was satisfactory. As for the important clinical feature-stage, we did not perform related analysis on it due to the incompleteness of the stage information. Further studies and data are needed to uncover the role of stage. We visualized the pseudogene signature and the other clinical information by a nomogram to simplify the use of this signature in clinical practice. Last, to further understand the biological function and explore the underlying oncogenic mechanism of the four pseudogenes, co-expression analysis was employed. Results showed the four pseudogenes were involved in multiple biological processes and pathways including malignant phenotype, immune, and DNA/RNA editing, which might be the underlying mechanism of osteosarcoma progression. Last, we compared the gene signature and pseudogene signature by ROC curve and found the pseudogene signature is a little better than the gene signature. The AUC of them were very close indicating the two signatures may have similar performance. Maybe the patient sample size leads to these results, a large size cohort is needed to verify this finding.

There are some limitations and shortcomings in this study that cannot be ignored. First, this study was mainly focused on data mining and data analysis, which are based on methodology and the results were not validated using experiments. Further experiments are needed to verify the findings of this study. Second, the datasets we were able to obtain were limited as we could only obtain one osteosarcoma dataset that contained both patient RNA-seq data and clinical follow-up information. If there were another dataset that matched our requirements, it could have been used to further validate our results. Additional datasets should be included to obtain a better result. Besides, there is currently no other study exploiting pseudogene signature for osteosarcoma, meaning that we also cannot validate our result in another independent study. Third, when constructing a pseudogene signature for prognosis, one must take it into consideration of the application of such a model. Since different methods of detecting pseudogenes might lead to different results, the procedure of detection, quantification, and determination of transcriptional activity of pseudogenes must be standardized [43]. Therefore, the four newly found prognosis-related pseudogenes deserve more attention and the next step for our research is to validate our results using experiments. We hope that these results could give other researchers inspiration for further study.

## 5. Conclusions

Taken together, we identified a novel four-pseudogene signature for osteosarcoma which is a promising independent survival predictor and served as an important biomarker for guiding the clinical treatment of osteosarcoma to improve management for patients. In addition, our findings provide new insights into exploring the underlying molecular mechanisms of osteosarcoma, and present a promising new prognostic marker. Therefore, our findings in the signature have a very promising clinical significance.

## Figures and Tables

**Table 1:** Clinical characteristics of patients in osteosarcoma cohort in this study.

	Alive (*n* = 57)	Dead (*n* = 37)	Total (*n* = 94)
Gender			
**FEMALE**	25	15	40
**MALE**	32	22	54
Age			
**Mean (SD)**	15.19 (5.75)	14.62 (4.40)	14.97 (5.27)
**Median (MIN, MAX)**	15 (3, 39)	14 (5, 32)	14.5 (3, 39)
Site			
**Leg/foot**	53	30	83
**other**	4	7	11
Metastatic status			
**Metastatic**	7	15	22
**Non-metastatic**	50	22	72

**Table 2:** 125 survival related pseudogenes screened by univariate cox regression analysis.

Pseudogene	HR	CI95	*p*-Value	Pseudogene	HR	CI95	*p*-Value
**RP4_706A16.3**	2.14	1.5–3.05	2.60 × 10^−5^	**RP11_271C24.3**	1.65	1.07–2.55	0.023498
**RP11_326A19.5**	0.57	0.43–0.76	0.000127	**NCF1C**	0.61	0.4–0.94	0.023523
**AC090602.2**	1.55	1.19–2.02	0.001094	**RP11_551L14.1**	0.64	0.43–0.94	0.023633
**RPL37P6**	1.52	1.18–1.97	0.001415	**RPL23AP53**	1.54	1.06–2.23	0.023792
**RP11_126D17.1**	1.61	1.19–2.17	0.00183	**ZNF815P**	0.53	0.31–0.92	0.024488
**NBPF2P**	1.84	1.25–2.71	0.001885	**AC002075.4**	1.41	1.04–1.91	0.025616
**RP11_25I15.1**	1.53	1.17–2	0.002111	**RPL7P47**	1.73	1.07–2.8	0.025737
**ZC3H11B**	1.72	1.21–2.44	0.002279	**CTC_451P13.1**	1.49	1.05–2.11	0.026269
**AP000936.1**	1.62	1.18–2.21	0.002517	**RP11_36C20.1**	1.81	1.07–3.07	0.026794
**RPS3AP44**	1.96	1.26–3.05	0.00281	**RP11_62J1.3**	0.54	0.31–0.93	0.026951
**SRSF9P1**	0.35	0.17–0.7	0.00321	**RPL7P9**	1.47	1.04–2.06	0.027115
**SLC25A24P1**	1.79	1.22–2.65	0.003255	**RP11_10G12.1**	1.45	1.04–2.01	0.027132
**CTD_2318O12.1**	1.58	1.15–2.16	0.004281	**EEF1A1P11**	1.44	1.04–1.99	0.027742
**RPS4XP16**	2.29	1.29–4.07	0.00472	**RPL13P12**	0.79	0.64–0.98	0.028947
**AB019441.29**	2.08	1.24–3.48	0.005308	**RP11_155G14.5**	1.47	1.04–2.08	0.029335
**CTA_963H5.5**	0.5	0.3–0.81	0.005308	**RPL13AP7**	1.57	1.04–2.37	0.030481
**ZNF37BP**	1.82	1.19–2.77	0.0055	**RP11_488L18.4**	1.47	1.04–2.08	0.030529
**EIF1AXP1**	1.31	1.08–1.58	0.005551	**TMSB4XP8**	0.75	0.57–0.97	0.031485
**RPL41P2**	1.59	1.14–2.22	0.005779	**FCF1P2**	0.6	0.38–0.96	0.031956
**PLA2G12AP1**	0.47	0.27–0.81	0.006369	**CSPG4P13**	1.35	1.03–1.78	0.03249
**RP11_23J18.1**	0.44	0.24–0.79	0.006523	**RPL41P1**	1.09	1.01–1.17	0.0327
**RP11_535M15.2**	1.83	1.18–2.83	0.006615	**RPL7P23**	1.75	1.05–2.93	0.032774
**PTGES3P1**	0.5	0.3–0.83	0.006904	**RP11_175B9.3**	1.32	1.02–1.69	0.033142
**RP11_16F15.2**	1.79	1.17–2.72	0.006917	**RP11_1166P10.1**	1.55	1.03–2.31	0.033608
**RPL10P3**	1.44	1.1–1.87	0.007168	**SRP68P3**	1.28	1.02–1.6	0.034297
**RP11_197B12.1**	1.41	1.1–1.81	0.007359	**RP11_51F16.9**	0.57	0.34–0.96	0.034541
**MTND4P12**	1.3	1.07–1.57	0.007377	**FAM86C2P**	2.07	1.05–4.08	0.034789
**RP11_120B7.1**	1.81	1.16–2.82	0.008388	**AC073850.6**	1.41	1.02–1.94	0.035167
**AC005077.14**	1.42	1.09–1.83	0.008434	**HTR7P1**	0.41	0.18–0.94	0.0355
**RP11_255H23.5**	1.78	1.16–2.73	0.008551	**CALM2P2**	1.41	1.02–1.95	0.035662
**RP11_494O16.3**	0.55	0.35–0.86	0.008747	**RP11_587D21.1**	1.34	1.02–1.76	0.035695
**RP1_95L4.4**	0.46	0.25–0.82	0.009153	**DSTNP2**	0.6	0.37–0.97	0.038699
**AC010468.1**	1.83	1.16–2.9	0.009748	**AC144530.1**	1.69	1.03–2.78	0.039254
**MST1L**	1.66	1.13–2.44	0.009936	**AC004967.7**	1.45	1.02–2.07	0.039533
**AC079781.5**	1.39	1.08–1.8	0.010811	**NCF1B**	0.68	0.46–0.98	0.039578
**HSP90AB2P**	1.87	1.15–3.04	0.011429	**RP11_381E24.1**	1.64	1.02–2.63	0.039904
**AC009474.2**	1.45	1.09–1.93	0.01172	**RPS3AP6**	1.46	1.02–2.08	0.040027
**CTB_75G16.1**	1.76	1.13–2.74	0.012065	**RPL35P2**	0.59	0.36–0.98	0.040168
**TMSB10P2**	0.64	0.45–0.91	0.012102	**RPL4P4**	2.04	1.03–4.04	0.040672
**RP3_342P20.2**	1.88	1.15–3.08	0.012513	**HLA_H**	0.77	0.6–0.99	0.040894
**HLA_U**	0.6	0.41–0.9	0.012586	**RPS15AP12**	1.76	1.02–3.04	0.041171
**RPL7P32**	1.69	1.12–2.55	0.01278	**AC141586.5**	1.57	1.02–2.44	0.041404
**RP11_302I18.1**	1.96	1.15–3.32	0.013077	**EEF1A1P12**	1.4	1.01–1.93	0.041698
**RP11_360D2.2**	1.39	1.07–1.81	0.013836	**ANKRD36BP2**	1.39	1.01–1.9	0.042281
**EEF1A1P1**	1.63	1.1–2.41	0.014059	**U47924.6**	0.67	0.45–0.99	0.042954
**RP11_20O24.1**	1.49	1.08–2.05	0.014879	**RP11_372E1.1**	1.72	1.02–2.89	0.042994
**RPS20P22**	1.44	1.07–1.95	0.015777	**RSL24D1P6**	0.56	0.32–0.98	0.043269
**RP11_583F2.6**	1.5	1.08–2.08	0.016158	**RPS26P31**	1.49	1.01–2.21	0.044184
**SMG1P1**	1.57	1.09–2.26	0.016217	**GGNBP1**	1.51	1.01–2.24	0.044241
**RP11_592N21.1**	1.55	1.08–2.22	0.016333	**FAM195CP**	0.67	0.45–0.99	0.044479
**SPATA20P1**	0.7	0.53–0.94	0.016488	**EEF1A1P9**	1.32	1.01–1.74	0.044928
**RP11_501C14.7**	0.55	0.34–0.9	0.016961	**CES5AP1**	1.25	1–1.55	0.045574
**RPL13AP6**	1.75	1.1–2.78	0.017922	**RP11_553P9.1**	1.61	1.01–2.57	0.045933
**RPS10L**	1.65	1.09–2.49	0.018544	**RP11_43F13.1**	1.53	1.01–2.34	0.046476
**CSAG4**	0.65	0.45–0.93	0.019462	**CTD_2192J16.15**	1.56	1.01–2.42	0.046513
**TOB2P1**	1.56	1.07–2.27	0.019856	**CH507_42P11.2**	0.67	0.45–0.99	0.046872
**RP11_344N17.15**	0.6	0.39–0.92	0.020105	**ADCY10P1**	1.5	1.01–2.25	0.047079
**MLLT10P1**	0.66	0.47–0.94	0.020725	**RP11_761N21.2**	1.33	1–1.76	0.04724
**ESPNP**	1.29	1.04–1.61	0.021376	**RP11_736N17.9**	1.29	1–1.66	0.047554
**RPL7AP28**	0.43	0.21–0.89	0.021683	**RP11_504P24.3**	1.43	1–2.04	0.047587
**RP11_241F15.10**	0.64	0.44–0.94	0.022711	**RPS26P5**	1.35	1–1.81	0.04809
**AC011737.2**	1.61	1.07–2.42	0.023174	**REXO1L1P**	1.45	1–2.1	0.048596
				**RPS11P5**	1.45	1–2.09	0.048755

**Table 3:** Results of multivariate cox regression analysis for the 15 pseudogenes screened by LASSO regression.

Characteristics	Hazard Ratio	CI95	*p*-Value	Significance
RP11_551L14.1	0.63	0.4–0.99	0.044	*
RPL7AP28	0.42	0.2–0.89	0.023	*
SRSF9P1	0.68	0.3–1.55	0.36	
ZC3H11B	1.29	0.85–1.96	0.235	
RP4_706A16.3	1.55	1–2.41	0.049	*
AC079781.5	1.16	0.84–1.6	0.372	
NBPF2P	1.56	0.86–2.82	0.14	
TMSB10P2	0.88	0.55–1.41	0.589	
ZNF815P	0.62	0.3–1.27	0.193	
SLC25A24P1	1.35	0.8–2.3	0.264	
CTA_963H5.5	0.71	0.37–1.37	0.311	
RP11_241F15.10	0.64	0.4–1.01	0.057	
RP11_23J18.1	0.91	0.46–1.78	0.773	
RP11_326A19.5	0.51	0.32–0.8	0.004	**
RP11_344N17.15	0.72	0.44–1.18	0.193	

**Table 4:** Score and clinical information of the osteosarcoma cohort.

	High Risk (47)	Low Risk (47)	Total (94)
Gender			
Female	23	17	40
Male	24	30	54
Age			
Mean (SD)	14.8 (5.27)	15.13 (5.36)	14.97 (5.29)
Median (min,max)	14 (5, 39)	15 (3, 32)	14.5 (3, 39)
Metastatic status			
Metastatic	15	7	22
Non-metastatic	32	40	72
Site			
Leg/foot	39	44	83
Other	8	3	11
Risk score			
Mean (SD)	5.55 (10.15)	0.47 (0.32)	3.01 (7.58)
Median (min,max)	2.53 (1.23, 66.4)	0.5 (0.01, 1.21)	1.23 (0.01, 66.4)
Status			
Alive	28	38	66
Dead	19	9	28

**Table 5:** A simple summary table for results of this article.

Total PG	Studied PG	Survival-Related PG	Signature-PG
14,126	1333	125	4
**3Y-AUC**	**5Y-AUC**	**8Y-AUC**	**KM-P-value**
Whole patients			
0.885	0.878	0.796	<0.0001
Gender subgroup (male)			
0.82	0.828	0.707	0.0064
Gender subgroup (female)			
0.965	0.947	0.947	<0.0001
Age subgroup (<18)			
0.902	0.888	0.788	<0.0001
Age subgroup (≥18)			
0.828	0.861	0.796	0.038
Metastatic subgroup (non-meta)			
0.871	0.87	0.743	0.00014
Metastatic subgroup (meta)			
0.91	0.89	0.89	<0.0001
Gene signature			
0.821	0.861	0.781	<0.0001
Combined signature			
0.937	0.956	0.871	<0.0001

PG: pseudogene; Y: year.

## Supplementary Materials

The following are available online at https://www.mdpi.com/2073-4425/10/6/414/s1, Table S1: The results of univariate analyses for the significant survival-related pseudogenes, Table S2: The detail of clinical information for the 94 osteosarcoma patients.

## Abbreviations

AUC	Area under curve
BP	Biological process
DAVID	Database for Annotation, Visualization, and Integrated Discovery
GO	Gene ontology
KEGG	Kyoto Encyclopedia of Genes and Genomes
K–M	Kaplan–Meier
OS	Overall survival
PCGs	Protein coding genes
ROC	Receiver operating characteristic
